# CAREGIVING FOR OLDER ADULTS: EXPLORING THE IMPLICATIONS FOR FAMILIAL RELATIONS, DYNAMICS, AND FRIENDSHIP

**DOI:** 10.1093/geroni/igad104.1215

**Published:** 2023-12-21

**Authors:** Arthur Chia, Rahul Malhotra

## Abstract

In this symposium, we discuss about various aspects of caregiving for older adults who require care because of chronic illness, frailty, and/or long-term disability. Their needs take many forms and so do the demands and concerns of their caregivers, comprising family members and close friends. Under these trying circumstances, relationships are put to the test; shared sensibilities and understandings built over decades of marital and family life begin to unravel, and friendships fray. Thus, caregiving has profound implications for the family as well as non-family members who provide care. Caregiving itself is marked by varying intensities expressed in anguished voices of loss, frustration, and anger but they also convey a deep sense of responsibility, gratitude, and love. There is much that caregiving could offer in terms of the potential for deeper meaning, connection, satisfaction, and goodness. Hence, the papers in this symposium will examine how caregiving responsibilities are distributed among family and non-family members; analyze the implications of caregiving on the notion of home/homeliness for caregivers; account for the meaning of caregiving for a parent or older family member as diverse expressions of filial piety; and explore the issues and challenges around caregiving for older adults with cognitive impairment including dementia from the perspectives of family and non-family members who are caregivers. Collectively, the papers highlight the relational and reciprocal nature of caregiving, and in which intimate relational and cultural processes shape not only the quality and experiences of caregiving but also foundations of the family and notions of home.

